# Risk factors affecting prognosis in metachronous liver metastases from WHO classification G1 and G2 gastroenteropancreatic neuroendocrine tumors after initial R0 surgical resection

**DOI:** 10.1186/s12885-019-5457-z

**Published:** 2019-04-08

**Authors:** Yang Lv, Xu Han, Xue-Feng Xu, Yuan Ji, Yu-Hong Zhou, Hui-Chuan Sun, Jian Zhou, Jia Fan, Wen-Hui Lou, Cheng Huang

**Affiliations:** 1 Department of Pancreatic Surgery, Zhongshan Hospital, Fudan University, 136 Yi Xue Yuan Rd, Shanghai, 200032 China; 2 Department of Pathology, Zhongshan Hospital, Fudan University, 136 Yi Xue Yuan Rd, Shanghai, 200032 China; 3 Department of Oncology, Zhongshan Hospital, Fudan University, 136 Yi Xue Yuan Rd, Shanghai, 200032 China; 4 Department of Liver Surgery and Transplantation, Liver Cancer Institute and Zhongshan Hospital, Fudan University, 136 Yi Xue Yuan Rd, Shanghai, 200032 China; 5 Key Laboratory for Carcinogenesis and Cancer Invasion, Chinese Ministry of Education, Beijing, China

**Keywords:** Gastroenteropancreatic neuroendocrine tumor, Metachronous liver metastasis, Nomogram, Prognosis

## Abstract

**Background:**

Here we describe the treatments and prognosis for metachronous metastases from gastroenteropancreatic neuroendocrine tumors (GEP-NETs) after initial R0 surgical resection at a large center in China.

**Methods:**

The clinicopathological data and survival outcomes for 108 patients (median age, 54.0 years) with metachronous hepatic metastatic GEP-NETs disease who were initially treated using R0 surgical resection between August 2003 and July 2014 were analyzed using one-way comparisons, survival analysis, and a predictive nomogram.

**Results:**

Fifty-five (50.9%) patients had pancreatic NETs and 92 (85.2%) had G2 primary tumors. For treatment of the hepatic metastases, 48 (44.4%) patients received liver-directed local treatment (metastasectomy, radiofrequency ablation, transcatheter arterial chemoembolization, etc.), 15 (13.9%) received systemic treatment (interferon, somatostatin analogs, etc.), and 45 (41.7%) received both treatments. Multivariable analyses revealed that OS was associated with hepatic tumor number (*P* < 0.001), treatment modality (*P* = 0.045), and elevated Ki-67 index between the metastatic and primary lesions (*P* = 0.027). The predictive nomogram C-index was 0.63.

**Conclusions:**

A higher Ki-67 index in metastases compared to primary tumor was an independent factor for poor prognosis. Local treatment was associated with prolonged survival of hepatic metastatic GEP-NET patients. Optimal treatment strategies based on clinicopathological characteristics should be developed.

## Background

Neuroendocrine tumors (NETs), once known as carcinoid tumors [1, 2], are a heterogeneous group of rare tumors with complicated clinical manifestations. Approximately 70% of NETs originate from the gastroenteropancreatic (GEP) system [3]. During the last 30 years, the frequency of diagnosis of NETs has significantly increased; NETs represent 2% of all GEP system tumors [4]. At present, the complete resection of the primary tumor(s) is the only effective method (or precondition) for potentially curative treatment of GEP-NETs, though more than 50% of these tumors are unresectable at diagnosis [5]. However, even when radical resection of the primary mass is performed, NETs can be progressive with local recurrence or distant metastasis. If metastasic disease is present, the most common location of distant metastasis is the liver [6].

The optimal treatment for metachronous neuroendocrine liver metastasis is still unknown and many modalities have been reported. Currently surgical resection, transcatheter arterial chemoembolization (TACE), radiofrequency ablation (RFA) and peptide peceptor- padionuclide pherapy (PRRT) are recommended treatments for hepatic metastatic GEP-NET disease [7]. Feasible systemic treatments include use of somatostatin analogs, interferon (IFN)-α, PRRT and chemotherapy; these treatments have different antitumor activities [8]. However, the specific treatment protocols which are precisely suitable for the treatment for selected groups of patients remain unconclusive.

In 2010, the Ki-67 proliferative index and mitotic count were included in the World Health Organization (WHO) classification as diagnostic and prognostic factors for NETs [9]. According to the latest WHO classification of digestive tumors and expert consensus [10], grade G1 and G2 tumors were identified as more differentiated and having low proliferation activity. However, the relatively indolent characteristics of these tumors are associated with lower rates of detection of distant metastases [11].

Because of the low incidence rates of GEP-NETs with liver metastasis, there are few reports about the biological and clinicopathological findings of NETs in the liver that are also associated with the GEP system. There are also few published results on associated risk factors. Most studies of malignant GEP-NET disease have been performed in Western countries; only a few have been performed in Asian countries. Thus we performed this study to reveal the biological characteristics and treatment outcomes of hepatic metastatic GEP-NETs in a referral center in China.

The nomogram is a reliable method to quantify risk by identifying key factors for prognosis prediction [12]. However, the clinical applications of the nomogram in hepatic metastatic GEP-NET patients have not been determined. We used the data collected to identify prognostic factors that affected overall survival (OS) time and to construct a nomogram for prediction of prognosis. Internal validation was also performed to determine the specific prognostic capability of the nomogram.

## Methods

### Patients

Patients with hepatic metastatic GEP-NETs who were treated at Zhongshan Hospital, Fudan University (Shanghai, China) from August 2003 to July 2014 were consecutively enrolled in the study. Informed consent was obtained by all the patients. The study protocol followed the ethical guidelines of the Declaration of Helsinki and was approved by the Ethical Committee of Zhongshan Hospital of Fudan University. All the recruited patients underwent radical resection of primary lesion(s). Patients were excluded if any of following conditions were present: presence of distant metastasis at the initial diagnosis or at less than 6 months after the initial diagnosis [13], synchronous malignancy in other organs, removal of the tumor with endoscopic techniques, and incomplete medical records.

The diagnoses for the primary and liver metastatic lesions were based on pathological morphology and immunohistochemical assessment of a surgical specimen or of an intraoperative biopsy or needle biopsy performed by experienced pathologists, according to the site of origin and WHO criteria. Tumor grade was assigned according to the latest European Neuroendocrine Tumor Society classification guidelines for NET grading and staging [14]. The pathological characteristics that were recorded as present or absent in the tumors were tumor number, tumor necrosis and texture, and lymph-node (during the resection of primary lesion) and neural invasion (metastasis). The Ki-67 index and mitotic count results were obtained from the pathological examination records for primary and metastatic lesions; the examinations were performed on formalin-fixed, paraffin-embedded tissues. The Ki-67 proliferative index was expressed as the percentage of Ki-67 positive cells in 2000 tumor cells within areas of the highest immunostaining using the MIB1 antibody (Dako, Denmark). The increase in value of the Ki-67 indices between the primary and metastatic lesions was also recorded and was regarded as an elevation in the Ki-67 index [15]. Histopathology slides were stained using the Bond-Max Leica autostainer (Leica Biosystems, United Kingdom). Antibody detection was performed using the biotin-free Bond Polymer Refined Detection System (DS9800; Leica Microsystems).

### Follow-up and treatments

The first follow-up was performed within 2 months after surgery. Subsequent follow-up cycles ranged from 3 to 4 months or shorter periods of time depending on clinical stage and whether tumor relapse or metastasis occurred. The treatment modalities for liver metastases depended on the liver nodule patterns. For the surgically resectable nodules, surgical resection and RFA were the first treatment choices. TACE was used for patients with unresectable nodules and confirmed liver metastasis. Systemic therapy was also used for patients with liver metastasis only and concurrent metastasis in other organs [16]. The overall survival (OS) and metastasis time data were also recorded. OS duration was calculated from the date of diagnosis of GEP-NETs until tumor-specific death or was censored at the patient’s last follow-up visit, metastatic survival was estimated from the date of hepatic metastasis to tumor-specific death and the last follow-up [17]. The time to relapse (TTR) duration was the interval from the date of the primary site operation until the diagnosis of liver metastasis [18]. Follow-up management was performed via outpatient clinics and telephone interviews. The interval of imaging examination such as CT or MRI was 6 months. The duration of follow-up was estimated from the date of diagnosis of liver metastasis to the patients last follow-up date.

### Statistics

Statistical analyses were performed using SPSS Statistics 22.0 for Windows (IBM Inc.). During comparisons among different subgroups, quantitative variables were compared using Student’s t-tests. Qualitative variables were compared using the chi-square test or Fisher’s exact test. The Kaplan-Meier method was used to estimate OS time. Median follow-up time was determined as the median number of all follow-up time. Log-rank tests were used to detect statistically significant differences between survival curves. Cox proportional hazard models were used to estimate the hazard ratio (HR) and 95% confidence interval (CI) for duration of survival. Cox proportional hazards regression was used for multivariable analyses of survival. All statistical tests were two-sided; a *P*-value < 0.05 was considered to indicate a statistically significant result.

A predictive nomogram was formulated based on the results of a multivariable analysis and using the package ‘rms’ in R version 3.3.0 (http://www.r-project.org/). Validation and discrimination of the nomogram was determined using the Harrell concordance index (C-index) as an index of model performance. Higher C-index values indicated better discrimination. A value of 0.5 defined no predictive discrimination; a value of 1.0 defined perfect discrimination of individuals with different outcomes.

## Results

### Clinical characteristics of patients with metastatic GEP-NETs

Table 1 presents the results for the baseline characteristics for the entire population. A total of 108 patients were enrolled in the study. The median age was 54.0 years (range, 36–81 years); there were 57 (60.6%) male patients. All patients were diagnosed with GEP-NETs. There were 17 patients with gastric NETs, 4 with duodenal NETs, 3 with small intestinal NETs, 5 with ascending colon NETs, 24 patients with rectum NETs in the rectum, and 55 patients with pancreatic NETs (pNETs). All the metastatic diseases were metachronous. All of the recruited patients experienced recurrent or metastatic disease, or both, after R0 resection of primary lesion. The results for the WHO grade classification for the primary site indicated that 16 (14.8%) patients had grade-1 disease and 92 (85.2%) patients had grade-2 disease. Besides, only 5 (4.6%) NETs patients was found coexisted extra-hepatic metastasis during the postoperative follow-up, including 3 patients of lung metastasis and 2 patients of bone metastasis.

**Table 1:** Baseline characteristics of 108 patients

Characteristics		Frequency	%
Age	Median	54	
	< 60	70	64.8%
	≥60	38	35.2%
Gender	Male	65	60.2%
	Female	43	39.8%
Origin	Pancreas	55	50.9%
	GI tract	53	49.1%
	Foregut	21	19.4%
	Midgut	8	7.4%
	Hindgut	24	22.2%
Primary tumor Grade^a^	G1	16	14.8%
	G2	92	85.2%

^a^The grade was a pathological result from the examination of the primary site according to the 2010 WHO grade consensus

### Patterns of treatment

All the patients underwent R0 resection (R0 resection was characterized as the resection performed with curative intent when no evidence of metastasis was identified) of the primary tumor. The types of surgical resections performed to remove the primary tumors were pancreatico-duodenectomy (27 patients), distal pancreatectomy (25 patients), resection of the rectum (24 patients), partial gastrectomy (13 patients), right colectomy (5 patients), and total gastrectomy (4 patients).

For treatment of hepatic metastatic disease, 48 patients received liver-directed local treatment including TACE, RFA and metastasectomy, 15 patients received systemic treatment only (i.g., IFN, somatostatin analogs, and chemotherapy), and 45 patients received both systemic and local treatment. Sixty-one (56.5%) patients underwent surgical resection of metastatic lesion(s). Among the 108 patients, 65 (60.2%) patients received TACE treatment and 55 (50.9%) underwent surgical resection followed by TACE treatment; this group included 30 patients with gastrointestinal NETs (GI-NETs) and 25 patients with pNETs; 32 (29.6%) patients received RFA therapy; this group included 22 patients with GI-NETs and 10 patients with pNETs. Medical treatment was generally based on the use of somatostatin analogs, which were administered in 15 patients with pNETs and 24 patients with GI-NETs. Systemic chemotherapy and molecular targeted treatment were performed in 35 patients and 37 patients, respectively. Seven patients with pNETs were treated with everolimus; two were treated with sunitinib.

### Survival and clinical prognostic factors for metastatic GEP-NETs

**Fig. 1:**
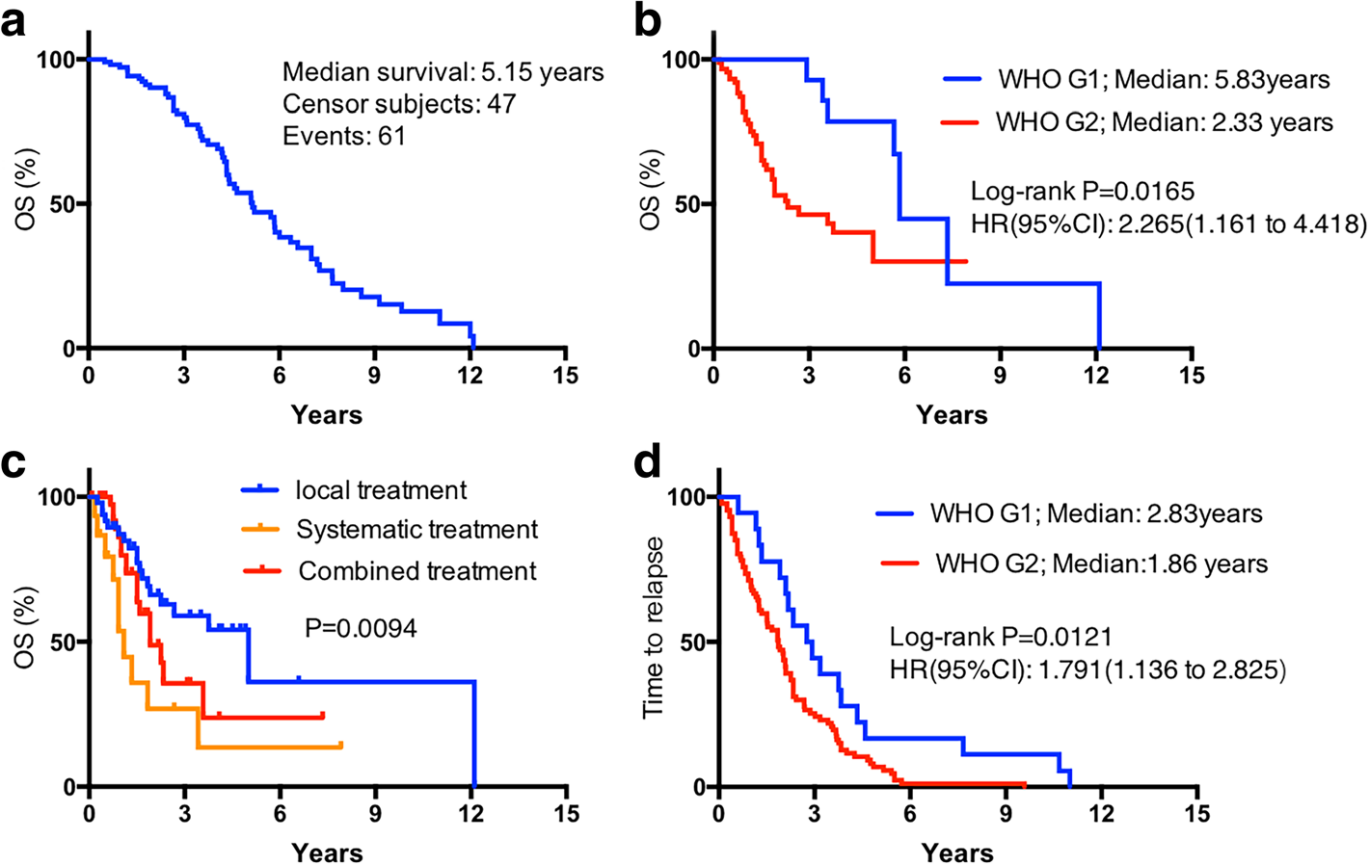
Overall survival (OS) and time to relapse (TTR) curves stratified by different variables in 108 patients with hepatic metastatic GEP-NET disease. OS curves stratified by all patients (**a**), WHO grade (**b**), treatment modality (**c**); TTR curves, by WHO grade (**d**)

The median follow-up duration was 36.8 months (range, 22.0–145.2 months, 95% CI: 30.2–50.6 months). Survival duration from diagnosis of GEP-NETs disease to tumor-specific death was 42.6 ± 38.1 months. Figure 1a presents the results for total duration of OS time for the 108 patients. The median survival duration was 5.15 years. Table 2 presents the results for metastatic survival duration according to clinicopathological characteristics. Survival was not significantly different by gender (*P* = 0.287), age (*P* = 0.818), primary tumor origin (*P* = 0.273), or longest hepatic tumor diameter (*P* = 0.343). OS was associated with number of liver metastases (*P* < 0.001) and lymph-node metastasis (*P* = 0.035). OS was also associated with primary tumor grade (Fig. 1b, grade-1 vs grade-2, 5.82 years versus 2.33 years, *P* = 0.017). Treatment modality (systemic treatment only, vs local treatment only vs combined treatments) was associated with OS (Fig. 1c, *P* = 0.009).

**Table 2:** Metastatic survival according to age, gender, and tumor characteristics (*n* = 108)

Characteristics		Median OS (Months)	95% CI	3-year Survival rate	*P*-value
Age	< 60	32.0	29.7–54.3	24.2%	0.818
	≥60	28.0	20.1–46.0	22.3%	
Gender	Male	22.8	6.2–39.4	15.4%	0.287
	Female	44.9	18.9–62.9	25.6%	
Primary tumor Grade	G1	70.0	40.2–98.1	21.74%	0.0028
	G2	28.0	18.7–36.1	6.25%	
Hepatic Tumor Number	Single	44.6	23.3–65.9	23.5%	0.000
	Multiple	13.0	9.5–16.6	5.3%	
Lymph-node meta	Present	32.0	11.3–52.7	11.3%	0.035
	Absent	53.0	10.4–75.6	23.3%	
Tumor origin	Pancreas	44.6	17.9–71.3	–	0.273
	GI tract	22.6	12.3–33.0	–	
Treatment modalities^a^	Local treatment	60.0	–	39.0%	0.009
	Systemic treatment	13.0	–	16.8%	
	Combined treatment	27.0	–	25.6%	
Longest hepatic tumor diameter	> 4.5 cm	32.0	12.1–51.8		0.343
	≤4.5 cm	39.7	29.9–71.4		

^a^The treatment modality was the treatment dealing with the hepatic metastatic masses

The median TTR duration for the total population was 28 months (95% CI, 24.1–35.8 months). The difference in TTR duration for gender (*P* = 0.514) and for tumor origin (*P* = 0.178) was not statistically significant. The association between WHO grade and TTR duration was statistically significant (Median survival duration: 2.83 years versus 1.86 years; *P* = 0.012; Fig. 1d).

### Pathological results and treatment outcomes for metastatic GEP-NETs

Samples of the hepatic lesions and related primary tumor tissues were taken from the excised specimen for analysis. Immunohistochemical staining was performed on primary tumor tissue samples from 92 patients. The Ki-67 index values were obtained from pathological examination of both the hepatic and related primary tumor tissues. The median ki-67 value of metastatic and primary was 22.8 and 5.9%, respectively. The positive rates of synaptophysin (Syn), neuron-specific enolase (NSE), and chromogranin A (CgA) were 72.3, 37.7, and 81.5%, respectively. The results for a comparison of immunohistochemical results for the primary site specimens between the GI-NETs group and pNETs group patients were summarized. The expressions of NSE and CgA were different between the two cohorts (*P* < 0.001 and *P* = 0.002, respectively). Our results also indicated that positive expression of CgA, NSE, or Syn in the primary tumor was not associated with OS time (*P* > 0.05).

### Univariable and multivariable analysis of metastatic survival duration in 108 patients

The results for the univariate and multivariable analyses of metastatic survival are presented in Table 3. The multivariable analysis of OS duration revealed that number of liver metastases (P < 0.001, hazard ratio [HR] = 0.07; 95% confidence interval [CI], 0.023–0.214), treatment modality (*P* = 0.045, HR = 0.801; 95% CI, 0.684–0.187, and HR = 0.922; 95%CI: 0.781–0.998), and elevation of the Ki-67 index (*P* = 0.027, HR = 1.396; 95% CI, 1.174–3.483) were statistically significant independent risk indicators for patient OS.

**Table 3:** Univariable and multivariable analyses of metastatic survival duration in 108 patients

Prognostic factors	Univariable analysis		Multivariable analysis	
	HR (95% CI)	*P*-value	HR (95% CI)	*P*-value
Age		0.818		0.361
<60	1.161 (0.575–2.344)		1.328 (0.722–2.442)	
≥60	1		1	
Gender		0.287		0.063
Male	1.375 (0.545–3.470)		0.511 (0.252–1.036)	
Female	1		1	
Primary tumor Grade		0.0028		0.293
G1	0.502 (0.144–0.756)		0.51 (0.147–1.782)	
G2	1		1	
Hepatic Tumor Number		0.000		0.000
Single	0.17 (0.123–0.214)		0.07 (0.023–0.214)	
Multiple	1		1	
Lymph-node meta		0.035		0.374
Present	1.057 (0.393–2.839)		0.988 (0.371–2.629)	
Absent	1		1	
Tumor origin		0.273		0.329
Pancreas	0.490 (0.139–1.732)		1.463 (0.682–3.138)	
GI tract	1		1	
Treatment modality		0.009		0.045
Local treatment	0.817 (0.690–0.917)		0.801 (0.684–0.887)	
Systemic treatment	0.921 (0.702–0.998)		0.922 (0.781–0.998)	
Combined treatment	1		1	
Elevation of ki67 index		0.022		0.027
Yes	1.997 (1.018–3.547)		1.396 (1.174–3.483)	
No	1		1	
Longest hepatic tumor diameter		0.343		0.775
> 4.5	0.644 (0.2–2.072)		0.875 (0.352–2.175)	
<=4.5	1		1	

**Fig. 2:**
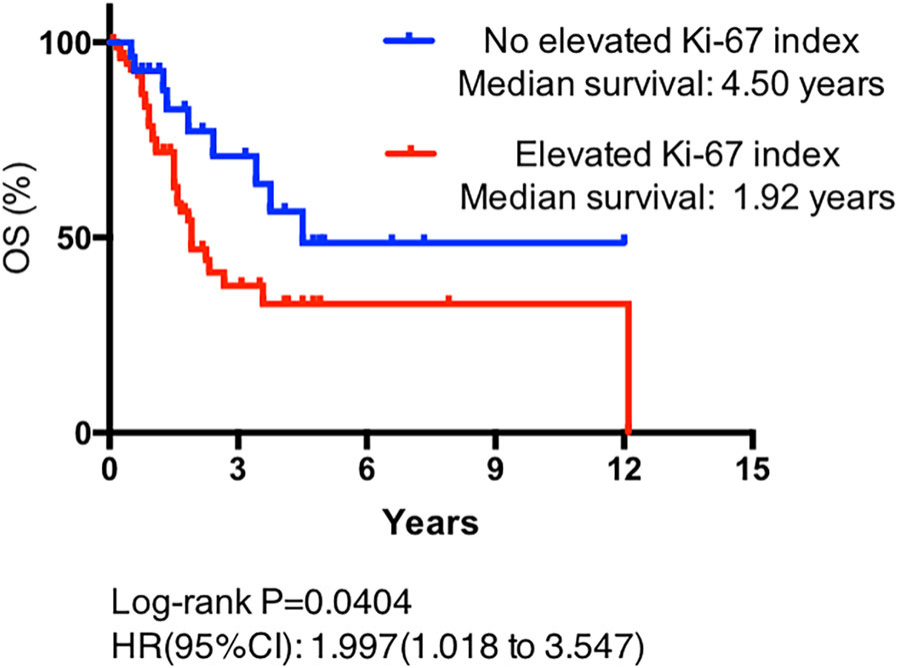
OS curves stratified by comparison of Ki-67 indices between hepatic and primary lesions in GEP-NET patients; an elevated Ki-67 index was associated with a shorter OS duration (*P* = 0.022)

The median Ki-67 index value was 5.0 (95% CI, 4.8–6.9) for the primary site and 20.0 (95% CI, 19.1–30.2) for the metastatic masses. Elevation of the Ki-67 index between the hepatic mass and the original mass was found in 57 (60.6%) patients. The GI-NET patient group had a higher elevated Ki-67 index incidence than the pNET patient group (41/53 vs 16/55, respectively, *P* = 0.015). The median OS time for the group of patients with no Ki-67 elevation was significantly longer compared with the group of patients with an elevated Ki-67 index (4.50 years vs 1.92 years, respectively, *P* = 0.022, Fig. 2).

### Construction and validation of the predictive nomogram for metastatic survival

**Fig. 3:**
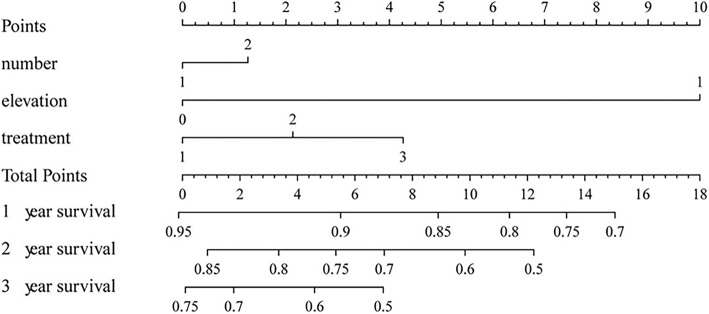
Nomogram to predict OS of patients with hepatic metastatic GEP-NETs. To validate the nomogram, the sum of each predictor point was charted on the total points axis. The estimated 1-, 2-, and 3-year OS rates were estimated by plotting a straight vertical line from the charted total points axis to the same OS rate axis. For the “number” line, 1 indicates a single hepatic lesion and 2 indicates multiple hepatic lesions. For the “treatment” line, 1, 2, and 3 refer to local treatment, combined treatment, and systematic treatment, respectively. For the elevation line, 0 indicates no elevation of the Ki-67 index

**Fig. 4:**
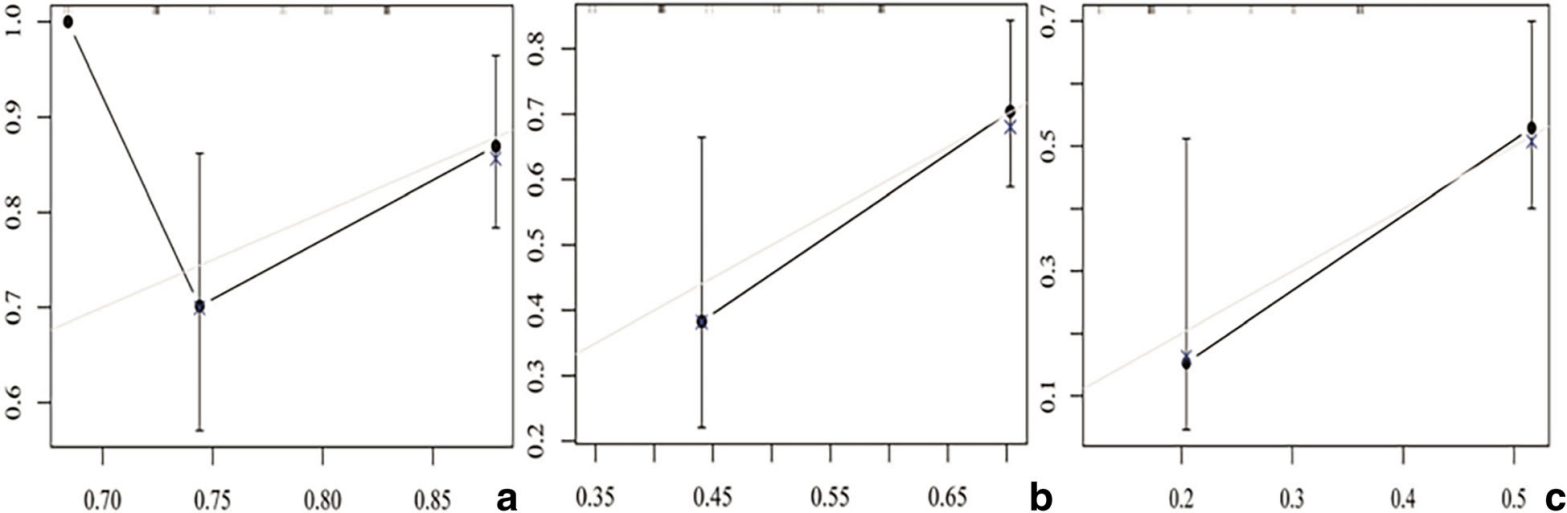
Calibration curve to predict overall survival of patients at 1 year (**a**), 2 years (**b**), and 3 years (**c**)

An accurate prognostic nomogram consisting of all significant independent prognostic factors (i.e., hepatic tumor number, treatment modality, and elevation of the Ki-67 index) for OS was constructed according to multivariable cox regression model results (Fig. 3). The C-index for OS prediction using the formulated nomogram was 0.63. A higher C-index value indicated better predictive accuracy for OS. The calibration plot for the probability of OS rates at 1-, 2-, and 3- years in our patients revealed good consistency between the observed OS and the nomogram-predicted OS (Fig. 4).

## Discussion

NETs have been substantially investigated and some of the findings have been applied to clinical practice [19, 20]. During the past decade, published results indicated that there is a trend towards increasing incidental diagnoses of GEP-NETs; hepatic metastatic tumors have a high prevalence among patients with GEP-NET disease [4]. Approximately 30–85% of patients are diagnosed with metachronous hepatic metastases during the postoperative follow-up period. In general, G1 or G2 tumors with intermediate- or well-differentiated lesions are common in GEP-NET patients. Among the GEP- NETs patients, simultaneous and heterogeneous hepatic metastases always occur, and the clinical characteristics of these metastases have been an important topic for researchers. Therefore, we enrolled 108 hepatic metastatic G1/G2 GEP-NET patients to investigate the clinical characteristics with respect to prognosis-related factors in well-differentiated metastatic GEP-NET disease.

In this study population, the origins of hepatic metastatic NETs were found in diverse locations in the GEP system; grade-2 disease was the most common (85.2%). Median metastatic survival time, which was calculated from the diagnosis of liver metastasis to the final follow-up or tumor-specific death is consistent with the other published trials [21]. Surgery remains an essential component of many phases of GEP-NET management. The improvements in comprehensive treatments for metastatic GEP-NETs are also associated with significant survival advantages [16]. The site of the primary NETs is an important prognostic feature of GEP-NETs [1, 22, 23]. However, for the different subgroups in our study, there were no statistically significant differences in the median TTR or OS duration among the GI-NET group and pNET group patients.

Study results indicate that WHO grade variation is associated with prognosis [11]. In our study population, OS was significantly affected by WHO grade. The median OS duration for grade-1 and grade-2 recruited patients were separately 5.82 years versus 2.33 years, (*P* = 0.017). The patients with grade-1 tumors had longer survival times, compared with patients with grade-2 tumors. However, higher grade predicts better response after systemic chemotherapy in some studies [17], but not in others. The median TTR duration after surgical resection of the primary site tumor was 28.0 months with all sites of origin combined; there was an apparent statistically significant difference between grade (Median survival duration: 2.83 years versus 1.86 years; *P* = 0.012).

Our study results also indicated that metastatic survival was associated with hepatic tumor number (*P* < 0.001) and lymph-node metastasis (*P* = 0.035). Particularly for the metastatic lesions, the multiplicity of the hepatic metastases was significantly associated with shorter survival time. This result indicated that a careful intraoperative search for multifocality using wide exposure and systematic intraoperative ultrasound use should be performed [9, 24, 25].

Patients received local or systemic treatment modalities, or both, for metastases. These treatments were given actively to the patients recruited in our study. Our results indicated that the patients who received local treatment only or combined treatments tended to have longer survival times, compared with the patients who received systemic treatment only. This result indicated that some types of local treatment modalities (e.g., metastasectomy, TACE [26], and radiotherapy) have an important role in the treatment of metastatic GEP-NET disease (*P* = 0.009). The results of a large retrospective study indicated the role of surgery for treatment of distant pancreatic NETs [27]. However, there have been few studies of metastatic GEP-NET disease that compare local with systemic treatment modalities or systemic with both treatment modalities using a randomized controlled trial design [28, 29].

We also performed immunohistochemical staining on the primary tumor specimen. Therefore, the clinical significance of some immunohistochemical markers was validated in our study. Our results indicated that the positive rates of NSE and CgA were statistically higher in the GI-NET group patients than in the pNET group patients (*P* < 0.001). However, we found no statistically significant differences between the duration of OS and the positive expression of Syn, NSE, or CgA. Because a high Ki-67 index was defined as a prognosis-related factor in GEP-NET patients, we conclude that in GEP-NETs hepatic metastatic lesions had higher Ki-67 index values, comparing to the primary site (*P* = 0.015). Our study revealed that elevation of the Ki-67 index was an independent prognostic factor for a shorter OS duration.

We used the data from the 108 cases to develop a nomogram to predict the 1-, 2-, and 3-year metastatic survival rates for patients with hepatic metastatic GEP-NETs, based on three significant factors (i.e., treatment modality, hepatic tumor number, and elevation of Ki-67 index). This approach effectively and visually predicted prognosis according to specific patient characteristics. The discrimination performance of the nomogram was evaluated using an internal bootstrap resampling method. Use of the C-index revealed the capability of the nomogram to predict 1-, 2-, and 3-year metastatic survival rates for patients with hepatic metastatic GEP-NETs.

This study had some limitations. First, our analysis was likely affected by limitations associated with all retrospective and single-center studies. However, as a very rare kind of tumor with metachronous liver metastasis, the cases number of our study were relatively large to have a significant result. What is more, grouping all GEP-NET tumors together may not be seen to be reasonable for clinical analysis owing to the different origins; however, the reclassification of all NETs according to the WHO 2010 scheme reduced the bias related to tumor origins. And we think that the homogeneous distribution of the combined prognostic factors in the GEP-NET group could reduce the bias related to the variable primary sites. Furthermore, development of novel treatment strategies is another issue, analyzing clinical data of patients receiving targeted therapy and SSA may not reach solid results, thus we consider analyzing the treatment modalities instead of analyzing such a small volume of treatment data solely. More detailed studies should be performed.

## Conclusions

In summary, we conclude that for hepatic metastatic GEP-NET disease, patients with lymph-node metastasis, multiple hepatic lesions, and higher primary tumor grade often achieved a short-term OS. The use of local treatment modalities was important for longer survival after treatment of metastatic GEP-NET disease. Elevation of Ki-67 index between the metastatic lesions and primary lesions was an important prognosis-related factor. The nomogram developed in this study revealed good discrimination capability to predict OS of hepatic metastatic GEP-NET patients.

## Abbreviations

CT: Computed tomography
GEP-NETs: Gastroenteropancreatic neuroendocrine tumors
MRI: Magnetic resonance imaging
OS: Overall survival
PRRT: Peptide peceptor- padionuclide pherapy
RFA: Radiofrequency ablation
SEER: Surveillance, Epidemiology, and End Results Program
TACE: Transcatheter arterial chemoembolization
TTR: Time to relapse
